# Effects of *in Utero* Exposure to Dicyclohexyl Phthalate on Rat Fetal Leydig Cells

**DOI:** 10.3390/ijerph13030246

**Published:** 2016-02-23

**Authors:** Xiaoheng Li, Xiaomin Chen, Guoxin Hu, Linxi Li, Huina Su, Yiyan Wang, Dongxin Chen, Qiqi Zhu, Chao Li, Junwei Li, Mingcang Wang, Qingquan Lian, Ren-Shan Ge

**Affiliations:** 1 The Second Affiliated Hospital & Yuying Children’s Hospital of Wenzhou Medical University, Wenzhou 325027, China; yaoyao_0111@126.com (X.L.); xminch@163.com (X.C.); lilinxi1234@163.com (L.L.); suhuina1988@163.com (H.S.); wyy19880119@163.com (Y.W.); gerenshan2@163.com (D.C.); flower3377@163.com (Q.Z.); dishboy@163.com (C.L.); 2 Department of Pharmacology, School of Pharmaceutical Sciences, Wenzhou Medical University, Wenzhou 325000, China; hgx@wzmc.edu.cn (G.H.); gravity172@gmail.com (J.L.); 3 Taizhou Hospital of Zhejiang Province, Taizhou 317000, China; wangmc@enzemed.com

**Keywords:** dicyclohexyl phthalate, fetal Leydig cell, Leydig cell aggregation, testosterone

## Abstract

Dicyclohexyl phthalate (DCHP) is one of the phthalate plasticizers. The objective of the present study was to investigate the effects of DCHP on fetal Leydig cell distribution and function as well as testis development. Female pregnant Sprague Dawley dams orally received vehicle (corn oil, control) or DCHP (10, 100, and 500 mg/kg/day) from gestational day (GD) 12 to GD 21. At GD 21.5, testicular testosterone production, fetal Leydig cell number and distribution, testicular gene and protein expression levels were examined. DCHP administration produced a dose-dependent increase of the incidence of multinucleated gonocytes at ≥100 mg/kg. DCHP dose-dependently increased abnormal fetal Leydig cell aggregation and decreased fetal Leydig cell size, cytoplasmic size, and nuclear size at ≥10 mg/kg. DCHP reduced the expression levels of steroidogenesis-related genes (including *Star*, *Hsd3b1*, and *Hsd17b3*) and testis-descent related gene *Insl3* as well as protein levels of 3β-hydroxysteroid dehydrogenase 1 (HSD3B1) and insulin-like 3 (INSL3) at ≥10 mg/kg. DCHP significantly inhibited testicular testosterone levels at ≥100 mg/kg. The results indicate that *in utero* exposure to DCHP affects the expression levels of fetal Leydig cell steroidogenic genes and results in the occurrence of multinucleated gonocytes and Leydig cell aggregation.

## 1. Introduction

Dicyclohexyl phthalate (DCHP) is one of a family of synthetic compounds known as phthalates. It is a diester formed by reaction of a molecule of phthalic acid with two molecules of cyclohexyl alcohol. It is widely applied to stabilize rubbers, resins, and polymers, including nitrocellulose, polyvinyl acetate, and polyvinyl chloride. In polyvinyl chloride products it is mainly used as a plasticizer, which increases polyvinyl chloride flexibility, transparency, durability, and longevity.

DCHP is not covalently bound to the polymers [1,2]. Instead, it is added to the polymers, and could easily leach out into the environment. DCHP has been detected in many foods, water, and the indoor environment [1,3]. Its monoester metabolite monocyclohexyl phthalate was also detected in human urine [4].

Several reports about the effects of DCHP on mammalian development and reproductive system were published [5,6,7]. Hoshino *et al.* investigated the two-generation effects of DCHP exposure at doses of 240, 1200, and 6000 ppm (about 14.3, 69.8, and 349 mg/kg) per day in the diet from gestational day (GD) 1–21 of F0 females in Sprague Dawley rats [6]. They found that the atrophy of seminiferous tubules of male F1 offspring occurred at 6000 ppm dose [6]. When Sprague Dawley rats were exposed *in utero* to DCHP at 250, 500, and 750 mg/kg per day by gavage from GD 6 to GD 20, male fetuses at GD 21 had low body weight at 750 mg/kg and reduced anogenital distance (AGD) at 500 and 750 mg/kg doses, suggesting that the anti-androgenic effect of DCHP, since AGD is the biomarker of androgen-dependent action [8]. Our previous study also demonstrated that DCHP directly inhibited the activities of two important Leydig cell androgen-biosynthetic enzymes, 3β-hydroxysteroid dehydrogenase 1 (HSD3B1, encoded by *Hsd3b1*) and 17β-hydroxysteroid dehydrogenase 3 (HSD17B3, encoded by *Hsd17b3*) [7], suggesting that DCHP exerts its action at least in part via suppression of many androgen-biosynthetic enzyme activities. Lower androgen activity is one of the typical manifestations of phthalate-mediated testicular dysgenesis syndrome [9]. Other phthalate-mediated manifestations of testicular dysgenesis syndrome include the abnormal aggregations of fetal Leydig cells and formation of multinucleated gonocytes (for a review see [10,11]). The term “testicular dysgenesis syndrome” was coined referring to a spectrum of reproductive disorders that originate in male fetal life [10]. Testicular dysgenesis syndrome includes cryptorchidism (undescended testes) and hypospadias (abnormal formation of the urethral meatus) in newborn boys and testicular cancer and reduced fertility in adult males [10]. The occurrence of multinucleated gonocytes (MNGs), abnormal fetal Leydig cell aggregation, decreased intratesticular testosterone levels, and reduced insulin-like factor 3 (*Insl3*) mRNA/protein (INSL3) expression in the fetal testis have been well documented in some phthalates including di(2-ethylhexyl) phthalate (DEHP) and di(2-butyl) phthalate (DBP) [9,12,13]. Therefore, these phthalate-mediated manifestions in rodents are similar to those of human testicular dysgenesis syndrome in human males, and are often referred as “phthalate syndrome” [10].

Testosterone and INSL3 are secreted by fetal Leydig cells in the male embryo. Fetal Leydig cells are important testicular endocrine cells, which are arranged usually in clusters with each cluster containing two or more fetal Leydig cells surrounded by several lawyers of collagen-rich membrane in the interstitial space between seminiferous tubules. Fetal Leydig cells are typically different from adult Leydig cells, the second generation of Leydig cells that emerge during pubertal period and do not appear in clusters. Fetal Leydig cells play important roles in the development of the male reproductive tract and the testis descent [10]. The number of fetal Leydig cells per cluster varies. A typical Leydig cell cluster showed the histological features with large round Leydig cells, which have many lipid droplets [14]. Although DCHP has been shown to affect mammalian development and reproductive system, its detailed effects on fetal Leydig cell development have not been well investigated. In the present study, we investigated the dose-dependent effects of DCHP on the fetal Leydig cell number, size, aggregation, specific gene expression levels as well as development of the fetal testis.

## 2. Materials and Methods

### 2.1. Animals and Treatment

Adult male and female Sprague-Dawley rats were purchased from the Shanghai Laboratory Animal Center (Shanghai, China). After acclimation for one week, male and female rats were mated. When the pregnancy was confirmed, the female rat was individually housed (23 ± 2 °C, relative humidity 55% ± 5%), in a 12-h light-dark cycle environment. The animals were housed in individually ventilated caging (IVC) cages (one rat per cage) on soft chip bedding and provided pellet chow (Shanghai Laboratory Animal Center). This study was approved by the Wenzhou Medical University Laboratory Animal Ethics Committee (Ethic Approval Number: wydw2015-0126), and all procedures were performed in accordance with the policies. The investigation conformed to the procedures described in the Guide for the Care and Use of Laboratory Animals published by the United States National Institutes of Health (NIH Publication No. 85-23, revised 1996). DCHP was purchased from Sigma (St. Louis, MO, USA) and suspended in corn oil (vehicle control) for gavage. The pregnant dams were gavaged with DCHP (Sigma) at 0 (control, corn oil), 10, 100, or 500 mg/kg body weight daily from GD 12 to GD 21 (*n* = 6 for each group). The choice of maternal exposure window from GD12 to GD21 is based on the fact that the fetal Leydig cells emerge at GD12 and a dam gives birth at GD21.5 [10]. Pregnant dams gave birth at GD 21.5. Following measurement of the pup body weight and length, and anogenital distance (AGD) of the male pups of each dam, the pups were put into the airtight IVC cages and were sacrificed by asphyxiation with CO_2_ at GD 21.5 (postnatal day 1). Three sets of randomly selected fetal testes (at least one testis per dam) were immediately removed, frozen in liquid nitrogen, and stored at −80 °C for further analysis of cell distribution (six testes per group), Leydig cell-specific mRNA levels (six testes per group), and testicular testosterone (six testes per group). Other remaining testes were fixed by Bouin’s solution for one day for histochemical staining of testes, which were used for analysis of testis dysgenesis (six testes per group), Leydig cell morphological changes (six testes per group), and semiquantation of Leydig cell specific protein levels (6 testes per group).

### 2.2. Testicular Testosterone Assay

Testicular steroids were extracted from the testes of control and DCHP-exposed pups (*n* = 6, control; *n* = 6, DCHP per dose) as described [13]. Testicular concentrations were measured by a tritium-based radioimmunoassay validated for use with rat antiserum, as described [13].

### 2.3. Immunohistochemical Staining for Desmin to Calculate the Frequency of Focal Testis Dysgenesis

In the fetal testis, the seminiferous tubules were surrounded by peritubular myoid cells. The peritubular myoid cell layer was brightly positive for desmin [15]. In the cross section of fetal testis, the seminiferous tubules were present as a smooth circle. The immunohistochemical detection of desmin was performed as described previously [16]. Briefly, testes were dehydrated in ethanol and xylene and embedded in paraffin for immunological analysis. Six testes per group were arrayed in a tissue array rack. 6 µm-thick transverse sections were prepared and mounted on glass slides (Cat. No. 12-550-15; Fisher Scientific Company, Hampton, NH, USA). Avidin–biotin immunostaining was performed using a kit (Vector Laboratories, Inc., Burlingame, CA, USA) according to the manufacturer's instructions. Antigen retrieval was carried out by boiling for 10 min in 10 mM (pH 6.0) citrate buffer, and endogenous peroxidase was blocked with 0.5% H_2_O_2_ in methanol for 30 min. The sections were then incubated with desmin antibody (ab32362, Abcam, Cambridge, UK) diluted 1:200 for 1 h at room temperature. The antibody-antigen complexes were visualized with diaminobenzidine alone, resulting in brown cytoplasmic staining in positively labeled peritubular cells. The sections were counterstained with Mayer hematoxylin, dehydrated in graded concentrations of alcohol, and cover-slipped with resin (Permount, SP15-100; Fisher Scientific). The testis showing at least one area of focal testis dysgenesis was referred as the focal dysgenic testis. The incidence of focal testis dysgenesis was calculated as the number of the focal dysgenic testis divided by the number of testes counted.

### 2.4. Hematoxylin and Eosin (HE) Staining for Multinucleated Gonocytes (MNGs)

Sections were stained using hematoxylin and eosin stain. To analyze the occurrence of MNGs, we used an Olympus BH-2 microscope (Olympus Optical, London, UK) to capture image. One complete testis cross-section from each animal was analyzed, and the percentage of seminiferous cord cross-sections that contained one or more MNGs was recorded and calculated as the percentage in total tubules counted.

### 2.5. 3β-HSD Enzymological Staining for Fetal Leydig Cells

3β-HSD is the biomarker of the fetal Leydig cell. Enzymological staining for 3β-HSD in the frozen section of the fetal testis was performed as described previously with a little bit modification [13]. In brief, frozen testes from four groups (0, 10, 100, and 500 mg/kg DCHP) were embedded in the same blocks as a set of tissue array. Ten-micrometer-thick cryostat sections were prepared at −20 °C. The incubation solution contained 0.4 mM etiocholanolone as substrate and 2 mM NAD^+^ as a cofactor. In addition, the incubation medium contained tetranitroblue tetrazolium as the H^+^ acceptor, and 0.1 M phosphate buffer at pH 7.2. The sections were incubated for 30 min at 37 °C in a humidified chamber in darkness. Additional sections were incubated without substrate as negative controls in order to determine nonspecific dehydrogenase effect. The sections were washed using phosphate buffered saline twice, and fixed using 4% phosphate saline buffered paraformaldehyde solution. After twice washes, the sections were counterstained using 4′6-diamidino-2-phenylindole (DAPI, Sigma). The sections were covered with 50% glycerol and covered with a coverslip. The morphology of the testis was observed using a fluorescence microscope (Olympus BH Series) at excitation wavelength 350 nm combined with bright-field photography. The number of fetal Leydig cell was counted by a stereological method as described [13]. In brief, to enumerate fetal Leydig cell numbers, 10 sections of each testis were randomly selected and fetal Leydig cells were counted. The total number of fetal Leydig cells per testis was calculated. Fetal Leydig cell clusters were counted, and frequency distributions were calculated as described [13].

### 2.6. Immunohistochemical Staining of 3β-HSD to Label Fetal Leydig Cells

The immunohistochemical detection of 3β-HSD was performed as above. The sections were then incubated with a 3β-HSD polyclonal antibody diluted 1:200 for 1 h at the room temperature. The antibody-antigen complexes were visualized with diaminobenzidine alone, resulting in brown cytoplasmic staining in positively labeled Leydig cells. The sections were counterstained with Mayer hematoxylin, dehydrated in graded concentrations of alcohol, and cover-slipped with resin (Permount, SP15-100; Fisher Scientific). Cell size and nuclear size were measured using image analysis software as below.

### 2.7. Computer-Assisted Image Analysis

Eight randomly selected fields in each of three nonadjacent sections per testis were captured using a BX53 Olympus microscope (Tokyo, Japan) equipped with digital camera interfaced to a computer. The images that were displayed on the monitor represented areas of 0.9 mm^2^ of testis. Cell size and nuclear size were estimated using image analysis software (Image-Pro Plus; Media Cybernetics, Silver Spring, MD, USA). Perimeters of the fetal Leydig cell and nucleus were drawn and calculated by the image analysis software. The cytoplasmic area was calculated by the area of fetal Leydig cell minus its nuclear area. More than 50 fetal Leydig cells were evaluated in each testis and the cell size and nuclear size and their ratio were averaged.

### 2.8. Real-Time PCR (qPCR)

Total RNA was extracted from rat testes in TRIzol according to the manufacturer’s instruction (Invitrogen, Grand Island, NY, USA). First strand synthesis and qPCR were performed as described [13]. Ribosomal protein S16 (*Rps16*) mRNA level was assayed in each sample as the internal control. The primers of seven other testicular genes were provided in Table S1. These genes including membrane receptor genes including luteinizing hormone receptor (*Lhcgr*); cholesterol transporting genes including cholesterol LDL receptor (*Scarb1*), steroidogenic acute regulatory protein (*Star*) and steroidogenic enzyme genes including CYP11A1 (*Cyp11a1*), 3β-HSD1 (*Hsd3b1*), P450c17 (*Cyp17a1*), 17β-hydroxysteroid dehydrogenase 3 (*Hsd17b3*) and INSL3 (*Insl3*). The relative mRNA levels of targeted genes were normalized to *Rps16* (internal control gene) using double standard curve method as described [17]. The gene symbols, GenBank accession No., and primer sequences are listed in Table S1.

### 2.9. Quantitative Immunohistochemical Staining of Leydig Cell Specific Proteins

Insulin-like 3 (INSL3) is an important hormone secreted by fetal Leydig cells to induce the testis descent [18]. 3β-HSD is the important androgen-biosynthesizing enzyme in the fetal Leydig cells [19]. Immunohistochemical staining of INSL3 or 3β-HSD was performed as was stated above. INSL3 or 3β-HSD staining was performed using rabbit monoclonal antibodies against INSL3 (AB6790, Abcam) and 3β-HSD (Abcam), respectively. Leydig cell cytoplasmic density and background area density were measured using image analysis software (Image-Pro Plus; Media Cybernetics) according to the manufacturer’s instruction. More than 50 fetal Leydig cells were evaluated in each testis and the density each samples was averaged.

### 2.10. Statistical Analysis

Values are expressed as mean ± SEM, and data were analyzed by using one-way ANOVA and then ad hoc Dunnett’s test to compare values from DCHP treated rats to control values. GraphPad Prism (Version 5 GraphPad Software, La Jolla, CA, USA) was used.

## 3. Results

### 3.1. General Reproductive Toxicology

The birth rate was defined as the number of pregnant rats that gave birth to pups/the number of female rats being pregnant as identified by the formation of the vaginal plug. The litter size each dam was defined as the number of pups that a dam gave birth to. When compared with the control, DCHP exposure did not affect the birth rate, the litter size, and the male-to-female sex ratio at GD 21.5 (Table 1). The body weight of the male pups was significantly reduced (*p* < 0.001) at ≥10 mg/kg doses of DCHP (Table 1). The AGDs of male pups at DCHP treatment at ≥100 mg/kg were also significantly shortened (*p* < 0.05).

### 3.2. Frequency of MNGs and Focal Testis Dysgenesis

**Figure 1:**
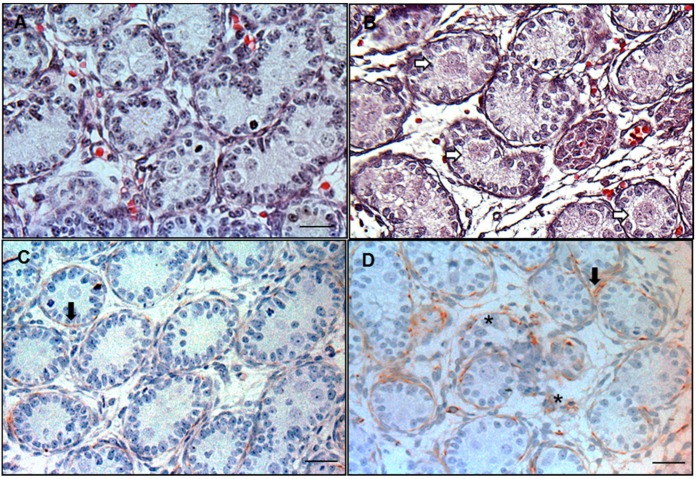
Occurrence of multinucleated gonocytes (MNGs) and focal testis dysgenesis in the representative photomicrographs of rat testis sections. (**A**) Control and (**B**) DCHP 500 mg/kg group for MNGs. Sections were stained with hematoxylin-eosin stains. White arrows point to MNGs in DCHP-treated testis; (**C**) Control and (**D**) 500 mg/kg group for the focal testis dysgenesis. Sections were stained immunologically for desmin. Black arrow points to the desmin staining (red color) for the normal shape of seminiferous tubules. Asterisk (*) points to the irregular and disintegrated seminiferous tubule, indicating the area of the focal testis dysgenesis. Scale bar = 25 μm.

Photomicrographs of testis showed that MNGs barely occurred in the testis of control group (Figure 1A), and less than 1% seminiferous cords in the control group contained MNGs (Table 2). DCHP dose-dependently resulted in an increase of occurrence of MNGs (Table 2), and the significant differences were detected at ≥100 mg/kg when compared to that of control. Desmin was immunologically stained in the testis sections from the control and each DCHP treatment group to visualize the integrity of seminiferous tubules. In the cross section of the control testis, seminiferous tubules were in regular round shape sounded with desmin-positive peritubular myoid cells (red color as showed by black arrow in Figure 1C), indicating that there was no focal testis dysgenesis area (Figure 1C). However, in the DCHP treated testis, irregular and distintegrated seminiferous tubules with desmin-positive peritubular myoid cells scattering in the interstitium (as showed by asterisk in Figure 1D) were formed, indicating that there is an increased incidence of focal testis dysgenesis in DCHP-treated testis. Of six testes in the 500 mg/kg DCHP group examined, three had at least one focal dysgenesis area (Figure 1D, Table 2). This suggests that DCHP treatment increases the occurrence of MNGs and focal testis dysgenesis.

### 3.3. Fetal Leydig Cell Numbers Per Testis and Leydig Cell Number and Size

**Figure 2:**
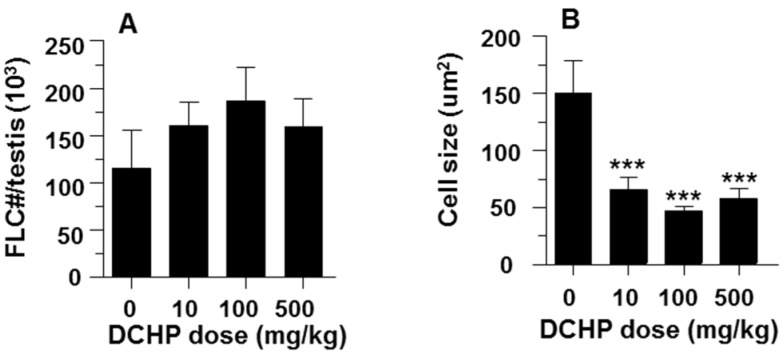

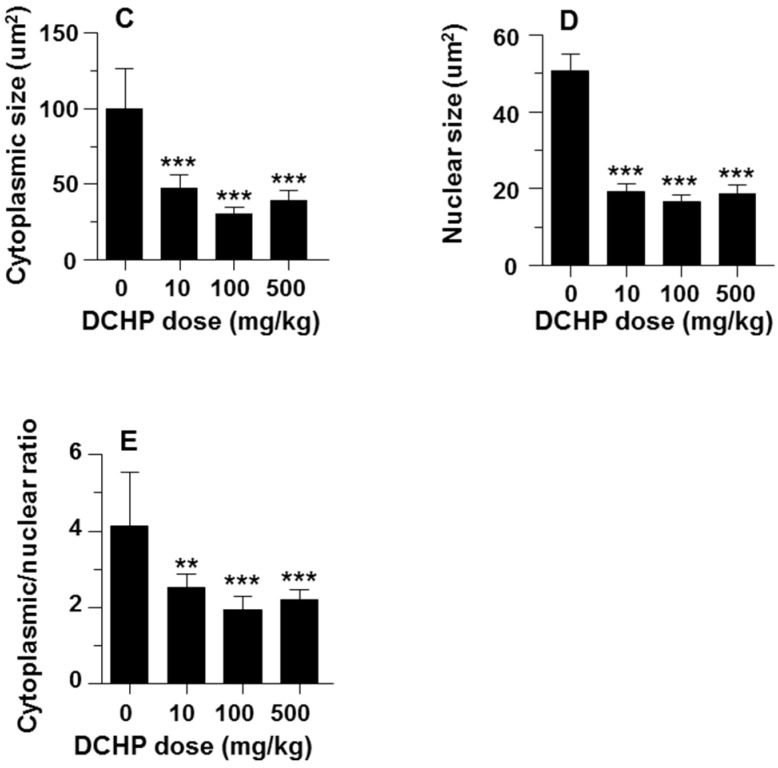
Effects of *in utero* DCHP exposure on fetal Leydig cell number, size, cytoplasmic size, nuclear size, and cytoplasmic/nuclear ratio. Pregnant dams were gavaged with 0 (control), 10, 100, and 500 mg/kg DCHP from GD 12 to GD 21. Measurements were performed at GD 21.5. Data are represented as mean ± SEM, *n* = 6 fetal testes. (**A**) Cell number per testis; (**B**) Cell size; (**C**) Cell cytoplasmic size; (**D**) Cell nuclear size; (**E**) Cell cytoplasmic/nuclear ratio. ** and *** indicate significant differences compared with control (DCHP 0 mg/kg) at *p* < 0.01 and *p* < 0.001, respectively.

Fetal Leydig cell numbers per testis did not change in all DCHP exposure groups (Figure 2A). The fetal Leydig cell size (Figure 2B), cytoplasmic size (Figure 2C), nuclear size (Figure 2D) and the ratio of cytoplasmic / nuclear size (Figure 2E) were significantly decreased even at 10 mg/kg DCHP.

### 3.4. Fetal Leydig cell distribution

**Figure 3:**
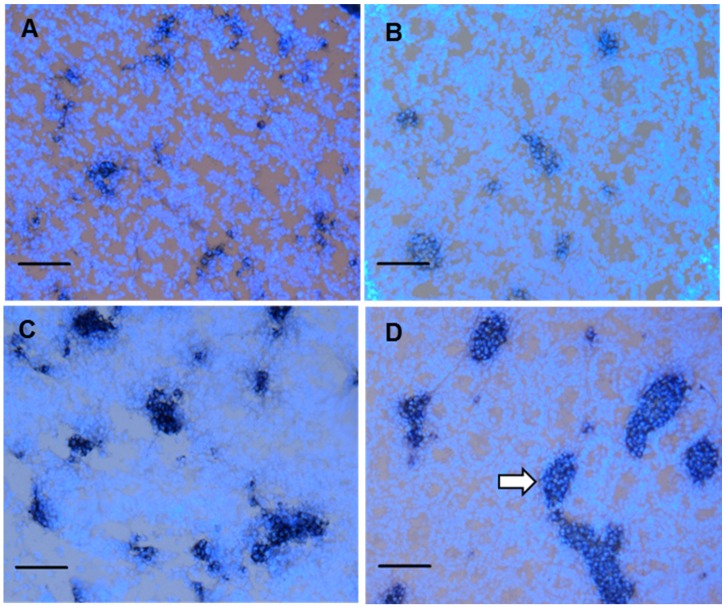
Effects of dicyclohexyl phthalate (DCHP) on fetal Leydig cell aggregation. Testis sections were stained by HSD3B1 for fetal Leydig cells and counterstained with DAPI for cell nuclei. Fetal Leydig cell clusters in the representative photomicrographs of testis sections: (**A**) 0; (**B**) 10; (**C**) 100; and (**D**) 500 mg/kg DCHP. Scale bar = 50 μm. White arrow points to a huge cluster of fetal Leydig cells after in utero exposure to DCHP.

Fetal Leydig cells are not uniformly distributed in the interstitial space of the fetal testis but rather are found in discrete clusters [14]. This was evident in micrographs (Figure 3). As shown in Table 3, the average fetal Leydig cell numbers per cluster increased in a dose-dependent manner at ≥10 mg/kg DCHP, and the percentage of large cell clusters (≥16 fetal Leydig cells/cluster) was significantly increased from control (1%) to 5%, 19%, and 28%, in the 10, 100, and 500 mg/kg DCHP groups, respectively. In the 500 mg/kg group, some clusters even contained more than 100 cells. In the control group, 74% ± 6% clusters contained 1–4 cells (Table 3). The percentage of the clusters containing 1–4 cells decreased significantly from the control to 66% ± 4%, 47% ± 6%, and 42% ± 5% in the 10, 100, and 500 mg/kg DCHP group, respectively. These data indicate that fetal Leydig cells trended to aggregate abnormally after exposure to ≥10 mg/kg DCHP.

### 3.5. Intratesticular Testosterone Level

Intratesticular testosterone concentrations at GD 21.5 were measured. As shown in Table 2, testicular testosterone levels trended lower with the increases of doses of DCHP and reached significant differences at ≥100 mg/kg.

### 3.6. Testicular Cell Gene Expression

**Figure 4:**
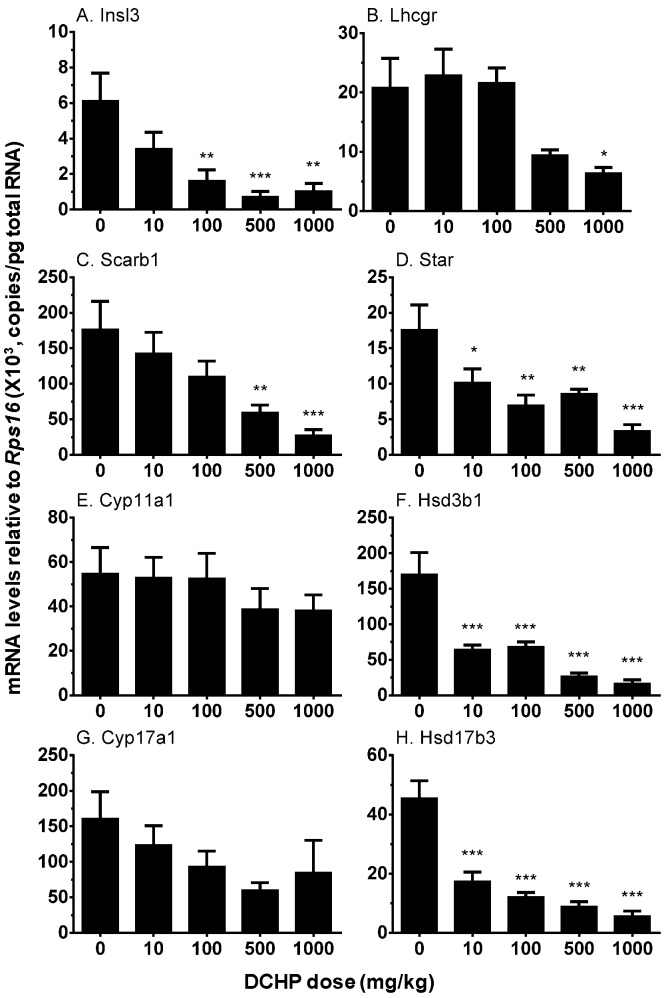
Real-time PCR analysis of mRNA levels in the testes from GD 21.5 after in utero DCHP exposure. Pregnant dams were gavaged with various doses of DCHP from GD 12 to GD 21. Data are presented as mean ± SEM (*n* = 6). The expression levels of Insl3 (**A**), Lhcgr (**B**), Scarb1 (**C**), Star (**D**), Cyp11a1 (**E**), Hsd3b1 (**F**), Cyp17a1 (**G**), and Hsd17b3 (**H**) were adjusted to that of Rps16. * *p* < 0.05; ** *p* < 0.01; *** *p* < 0.001, compared to control (0 mg/kg DCHP).

A set of mRNA transcripts were selected to examine the effects of DCHP on their expression levels. Those includes *Insl3* (encoding a protein, INSL3, in induction of testis decent), *Lhcgr* (encoding LHCGR for the response of LH stimulation to set up signaling of inducing steroidogenesis), *Scarb1* (encoding LDL receptor for the transporting circulating cholesterol into the fetal Leydig cells), *Star* (encoding a protein to transport cytosolic cholesterol into mitochondrial inner membrane for steroid synthesis), and androgen biosynthetic enzymes (*Cyp11a1*, *Cyp17a1*, *Hsd3b1*, and *Hsd17b3*). DCHP dose-dependently inhibited the expression of *Insl3*, *Star*, *Hsd3b1*, and *Hsd17b3* at ≥10 mg/kg. At 500 mg/kg, DCHP also inhibited the expression of *Lhcgr, Scarb1* and *Cyp17a1* (Figure 4). However, DCHP did not affect *Cyp11a1* expression.

### 3.7. Protein Levels of INSL3 and HSD3B1

**Figure 5:**
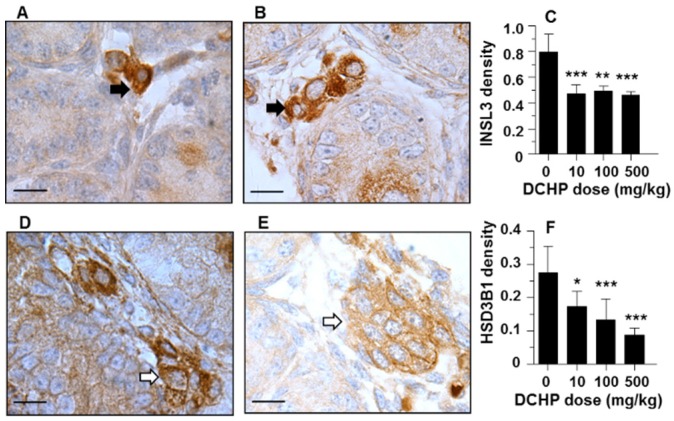
Semi-quantitative immunohistochemical analyses of INSL3 and HSD3B1 protein levels. (**A**, **D**), control group; (**B**, **E**) 500 mg/kg DCHP group. (**A**, **B**) INSL3; and (**D**, **E**) HSD3B1. Relative densities of INSL3 and HSD3B1 in the testis from GD21.5 after *in utero* DCHP exposures were compared to those of control (**C**, **F**). Black arrow: INSL3; white arrow: HSD3B1. Mean ± SEM, *n* = 6. * *p* < 0.05; ** *p* < 0.01; *** *p*< 0.001.

Immunohistochemical staining of INSL3 or HSD3B1 was performed on the testis of GD 21.5 (Figure 5). Semi-quantitative analysis revealed that DCHP reduced the protein expression of INSL3 and HSD3B1 at ≥10 mg/kg exposure groups, confirming the changes of their mRNA levels.

## 4. Discussion

In the present study, Sprague Dawley rats were administered orally with daily doses of DCHP from GD 12 to 21 with 10, 100, and 500 mg/kg. It remarkably induced testis dysgenesis syndrome, including the formation of focal testis dysgenesis, multinucleated gonocytes, and abnormal cluster of fetal Leydig cells, and the decreases of the expression levels of steroidogenesis-related genes (*Lhcgr, Star, Cyp11a1, Hsd3b1, Cyp17a1,* and *Hsd17b3*) as well as testis decent related gene *Insl3*.

DCHP increased the incidence of MNGs and focal testis dysgenesis after *in utero* exposure. The doses to induce of MNGs and focal testis dysgenesis required at least ≥100 mg/kg. This has been reported by other studies focused on the phthalates including DEHP and DBP [13,20]. Fetal Leydig cells were aggregated rather than dispersed when the doses of DCHP were increased. Previous studies showed similar results for *in utero* high-dose exposures to other phthalates including DEHP or DBP [12,13,21]. When compared to DEHP and DBP [12,13,21], DCHP showed similar potency to cause the aggregation of fetal Leydig cells (Table 3). Further analysis has suggested that the increased fetal Leydig cell number per cluster in response to DCHP does not result from the increasing of Leydig cell numbers (Table 3). In the current study, a low dose (10 mg/kg per day) *in utero* exposure for 10 days (GD 12–21) elicited fetal Leydig cell aggregation in the DCHP treated testis. The physiological consequences of fetal Leydig cell aggregation per se are uncertain, although there is evidence that it is associated with reduced fertility [22] and increased incidence of cryptorchidism [23]. The huge fetal Leydig cell cluster occurred in higher doses of DCHP unlikely seems Leydig cell tumors, because the cell numbers in response to DCHP did not change. The mechanism by which fetal Leydig cells become aggregated in huge clusters after DCHP exposure is not well understood. One possible theory is that these cells are initially more diffusely distributed but then form aggregated cluster under the influence of local growth factors. In our previous study, the expression levels of many growth factors in the testis were observed to be significantly increased in another phthalate DEHP exposure [13]. The mechanism of DCHP-induced occurrence of testis dysgenesis, multinucleated gonocytes and abnormal fetal Leydig cell aggregation is still unclear. It seems not related with the reduced testosterone production induced by phthalates [24].

DCHP *in utero* exposure also significantly reduced testicular testosterone levels (Table 2). Testosterone biosynthesis in fetal Leydig cells of the rat peaks on GD 19 [25]. The testosterone production during this period is essential for the development of male reproductive tract. Apparently, DCHP significantly reduced the expression levels of many steroidogenesis-related genes that include *Star* and *Hsd3b1* at the dose of as low as 10 mg/kg/day. When examining HSD3B1, the *Hsd3b1* gene product, we also found that DCHP significantly reduced HSD3B1 level at ≥10 mg/kg (Figure 5F).

Interestingly, the expression level of *Hsd17b3,* the last step of testosterone biosynthetic enzyme, which catalyzes androstenedione into testosterone, was also significantly suppressed by DCHP at as low as 10 mg/kg per day. In the rodent fetal testis, this enzyme is primarily expressed in Sertoli cells [26].

Fetal Leydig cells also produce INSL3, a peptide hormone that binds to the relaxin/insulin-like family peptide receptor 2 (RXFP2) in the gubernaculum. INSL3 acts on the gubernaculum to cause its shortening, thus inducing fetal testis descent [18,27]. Thus, disruption with the development of fetal Leydig cells may be one cause of cryptorchidism. The incidence of cryptorchidism has been documented to be significantly increased during the neonatal period after *in utero* exposures to other phthalates including DEHP and DBP [20,23,28,29]. The present study demonstrates that DCHP resulted in reduced INSL3 levels at as low as 10 mg/kg/day (Figure 5). DCHP also significantly reduced the expression levels of *Insl3* (Figure 4A).

## 5. Conclusions

These results, taken together, indicate that fetal exposures to DCHP have affected on fetal Leydig cell number, distribution, and most importantly, steroidogenic capacity.

## Figures and Tables

**Table 1:** Reproductive parameters after exposure to dicyclohexyl phthalate (DCHP) for 10 days.

Parameters	DCHP, mg/kg Per Day			
	0	10	100	500
Number of dams	6	6	6	6
Litter size	13 ± 1	13 ± 3	16 ± 1	12 ± 3
Birth rate	6/6	6/6	6/6	6/6
Pup male, %	49 ± 12	52 ± 15	59 ± 11	49 ± 9
Number of pups	37	44	51	30
Body weight, g	7.7 ± 0.7	6.5 ± 0.4 ***	6.4 ± 0.9 ***	6.5 ± 0.7 ***
AGD, mm	3.3 ± 0.3	3.0 ± 0.5	2.7 ± 0.2 *	2.6 ± 0.2 *

Dams of Sprague-Dawley rats were gavaged with DCHP from GD 12 to GD 21. Values are mean ± SEM. * *p* < 0.05, *** *p* < 0.001, compared to control (DCHP, 0 mg/kg).

**Table 2:** Occurrence of the focal testis dysgenesis, multinucleated gonocytes (MNGs) and testicular testosterone (T) levels after exposure to different doses of dicyclohexyl phthalate (DCHP) for 10 days.

Parameters	DCHP, mg/kg Per Day			
	0	10	100	500
Number of testes	6	6	6	6
Testis dysgenesis	0/6 ^a^	0/6	1/6	3/6
MNGs#/Tubule (%)	0.37 ± 0.24	2.08 ± 0.46	15.67 ± 2.70 ***	27.06 ± 2.90 ***
Testicular T, ng/mg	1.90 ± 0.25	1.71 ± 0.35	1.18 ± 0.23 *	0.62 ± 0.14 **

Dams of Sprague-Dawley rats were gavaged with DCHP from GD 12 to GD 21. Values are mean ± SEM, *n* = 6. ^a^ Testis number containing focal testis dysgenesis/testis was exampled; *, **, *** represent significant difference at *p* < 0.05, *p* < 0.01, *p* < 0.001, respectively, compared to control (DCHP, 0 mg/kg).

**Table 3:** Frequency distribution of cluster sizes of fetal Leydig cells after *in utero* exposure to dicyclohexyl phthalate (DCHP) for 10 days.

Cell No. Per Cluster	Frequency (%); DCHP, mg/kg Per Day			
	0	10	100	500
1–4	74 ± 6	66 ± 4 ***	47 ± 6 ***	42 ± 5 ***
5–8	18 ± 5	17 ± 2	17 ± 4	14 ± 5
9–16	7 ± 2	11 ± 1 **	17 ± 3 ***	16 ± 2 ***
>16	1 ± 1	5 ± 2 ***	19 ± 7 ***	28 ± 6 ***
Average	3 ± 0	5 ± 1 ***	11 ± 3 ***	13 ± 2 ***

Mean ± SEM, *n* = 6, ** *P* < 0.01, *** *P* < 0.001, respectively, compared to control (0 mg/kg).

## Supplementary Materials

The following are available online at www.mdpi.com/www.mdpi.com/1660-4601/13/3/246/s1, Table S1: Primers used in the Q-PCR analysis of this study.
