# Supplementary material for: Effects of in Utero Exposure to Dicyclohexyl Phthalate on Rat Fetal Leydig Cells
Source: Int J Environ Res Public Health. 2016 Feb 23;13(3):246. doi: 10.3390/ijerph13030246 (PMC4808909; doi:10.3390/ijerph13030246)
Supplement: Supplementary file 1 [file ijerph-13-00246-s001.pdf]

Effects of *in Utero* Exposure to Dicyclohexyl Phthalate on Rat Fetal Leydig Cells

Xiaoheng Li [1,†], Xiaomin Chen [1,†], Guoxin Hu [2], Linxi Li [1], Huina Su [1], Yiyan Wang [1], Dongxin Chen [1], Qiqi Zhu [1], Chao Li [1], Junwei Li [2], Mingcang Wang [3], Qingquan Lian [1,*] and Ren-Shan Ge [1,*]

Table S1. Primers used in the Q-PCR analysis of this study.

Gene Symbol	GenBank Accession NO.	Forward Primer	Reverse Primer
Rps16	X17665	5′AAGTCTTCGGACGCAAGAAA3′	5′TGCCCAGAAGCAGAACAG3′
Lhcgr	NM_012978	5′CTGCGCTGTCCTGGCC3′ Wrong:	5′CGACCTCATTAAGTCCCCTGAA3′
Scarb1	NM_031541	5′ATGGTACTGCCGGGCAGAT3′	5′CGAACACCCTTGATTCCTGGTA3′
Star	NM_031558	5′CCCAAATGTCAAGGAAATCA3′′	5′AGGCATCTCCCCAAAGTG3′
Cyp11a1	NM_017286	5′AAGTATCCGTGATGTGGG3′	5′TCATACAGTGTCGCCTTTTCT3′
Hsd3b1	NM_017265	5′CCCTGCTCTACTGGCTTGC3′	5′CCCTGCTCTACTGGCTTGC3′
Cyp17a1	NM_012753	5′TGGCTTTCCTGGTGCACAATC3′	5′TGAAAGTTGGTGTTCGGCTGAAG3′
Hsd17b3	NM_054007	5′ TTTCTTCGGGAGTAGGGGTTC 3′	5 TCATCGGCGGTCTTGGTCG3′
Insl3	NM053680	5′GTGGCTGGAGCAACGACA3′	5′AGAAGCCTGGTGAGGAAGC3′

© 2016 by the authors; licensee MDPI, Basel, Switzerland. This article is an open access article distributed under the terms and conditions of the Creative Commons by Attribution (CC-BY) license (http://creativecommons.org/licenses/by/4.0/).
